# Folic Acid Protects against Lipopolysaccharide-Induced Preterm Delivery and Intrauterine Growth Restriction through Its Anti-Inflammatory Effect in Mice

**DOI:** 10.1371/journal.pone.0082713

**Published:** 2013-12-06

**Authors:** Mei Zhao, Yuan-Hua Chen, Xu-Ting Dong, Jun Zhou, Xue Chen, Hua Wang, Shu-Xian Wu, Mi-Zhen Xia, Cheng Zhang, De-Xiang Xu

**Affiliations:** 1 Department of Toxicology, Anhui Medical University, Hefei, Anhui, China; 2 Anhui Provincial Key Laboratory of Population Health & Aristogenics, Hefei, Anhui, China; 3 School of Nursing, Anhui Medical University, Hefei, Anhui, China; 4 Department of Histology and Embryology, Anhui Medical University, Hefei, Anhui, China; 5 College of Life Sciences, Anhui Medical University, Hefei, Anhui, China; Medical Faculty, Otto-von-Guericke University Magdeburg, Medical Faculty, Germany

## Abstract

Increasing evidence demonstrates that maternal folic acid (FA) supplementation during pregnancy reduces the risk of neural tube defects, but whether FA prevents preterm delivery and intrauterine growth restriction (IUGR) remains obscure. Previous studies showed that maternal lipopolysaccharide (LPS) exposure induces preterm delivery, fetal death and IUGR in rodent animals. The aim of this study was to investigate the effects of FA on LPS-induced preterm delivery, fetal death and IUGR in mice. Some pregnant mice were orally administered with FA (0.6, 3 or 15 mg/kg) 1 h before LPS injection. As expected, a high dose of LPS (300 μg/kg, i.p.) on gestational day 15 (GD15) caused 100% of dams to deliver before GD18 and 89.3% of fetuses dead. A low dose of LPS (75 μg/kg, i.p.) daily from GD15 to GD17 resulted in IUGR. Interestingly, pretreatment with FA prevented LPS-induced preterm delivery and fetal death. In addition, FA significantly attenuated LPS-induced IUGR. Further experiments showed that FA inhibited LPS-induced activation of nuclear factor kappa B (NF-κB) in mouse placentas. Moreover, FA suppressed LPS-induced NF-κB activation in human trophoblast cell line JEG-3. Correspondingly, FA significantly attenuated LPS-induced upregulation of cyclooxygenase (COX)-2 in mouse placentas. In addition, FA significantly reduced the levels of interleukin (IL)-6 and keratinocyte-derived cytokine (KC) in amniotic fluid of LPS-treated mice. Collectively, maternal FA supplementation during pregnancy protects against LPS-induced preterm delivery, fetal death and IUGR through its anti-inflammatory effects.

## Introduction

Lipopolysaccharide (LPS) is the major component of the outer membrane of Gram-negative bacteria [1]. Humans are constantly exposed to low levels of LPS through bacterial infection. LPS is also widely present in the digestive tracts of humans and animals [2]. Gastrointestinal inflammatory diseases and excess alcohol intake can impair the intestinal barrier function, which might enhance translocation of LPS into the peripheral circulation [3]. LPS has also been detected in the cervical mucus and vaginal fluid from pregnant women with bacterial vaginosis [4]. According to a report from a meta-analysis, bacterial vaginosis doubled the risk of preterm delivery in pregnant women [5]. Microbiological studies suggest that intrauterine infection might account for 25%-40% of preterm delivery [6]. Moreover, mimicking maternal infection by exposing pregnant mice to LPS at late gestational stages caused preterm delivery, fetal death and intrauterine growth restriction (IUGR) [7-10]. Currently, antibiotic therapy is recommended regimen for pregnant women with bacterial infection including bacterial vaginosis [11]. However, increasing evidence has demonstrated that antibiotic therapy can not effectively prevent injection-associated preterm delivery [12,13]. Actually, antibiotics alone might cause impairment on the development of embryos and fetuses [14,15]. 

Indeed, LPS-induced inflammatory response contributes to infection-associated preterm delivery. Prostaglandins (PGs) have been demonstrated to be important mediators of LPS-induced fetal death and preterm delivery [16]. According to an earlier report, cyclooxygenase (COX)-2-mediated PGs production is a key pathophysiologic event in LPS-induced fetal death [17]. Celecoxib and SC-236, the selective COX-2 inhibitors, prevented LPS-induced fetal death and preterm delivery [18,19]. On the other hand, maternal LPS exposure resulted in the increase of inflammatory cytokines in maternal serum, amniotic fluid and placenta [20,21]. Moreover, pentoxifylline, an inhibitor of TNF-a synthesis, prevented LPS-induced fetal death and IUGR [8]. 

Folic acid (FA) is a water-soluble B-complex vitamin. The crucial roles of FA in one-carbon metabolism for physiological DNA synthesis and cell division have been convincingly demonstrated [22]. Abundant evidence demonstrates that FA deficiency during pregnancy is a cause of neural tube defects (NTDs) and FA supplementation reduces the risk of NTDs [23-26]. Recently, two cohort studies suggested that supplementation with FA during pregnancy may be protective against preterm delivery and low birth weight [27,28]. However, the mechanism underlying the association between FA and preterm delivery is obscure. A recent study suggested that FA might act by rectifying genital tract inflammatory milieu to reduce the risk of preterm delivery [29]. Actually, increasing evidence indicates that FA has an anti-inflammatory effect [30]. Hence, further studies to elucidate the role of FA against inflammation-related preterm delivery and IUGR are necessary. 

The aim of this study was to investigate the effects of maternal FA supplementation on LPS-induced preterm delivery, fetal death and IUGR in mice. Our results showed that FA supplementation alleviated LPS-induced preterm delivery, fetal death and IUGR. We demonstrated for the first time, to our knowledge, that FA-mediated protection against LPS-induced preterm delivery, fetal death and IUGR might be, at least partially, attributed to its anti-inflammatory effects. 

## Materials and Methods

### Chemicals and reagents

Lipopolysaccharide (Escherichia coli LPS, serotype 0127:B8) and FA were purchased from Sigma Chemical Co. (St. Louis, MO, USA). Nuclear factor-kappa B p65 (NF-κB p65, SC-372), phospho-inhibitor of kappa B (p-IκB, Ser 32, SC-8404), COX-2 (SC-1747-R), α-tubulin (SC-5286) and Lamin A/C (SC-6215) antibodies were from Santa Cruz Biotechnologies (Santa Cruz, CA，USA). β-actin antibody was from Boster Bio-Technology Co. LTD (Wuhan, China). Chemiluminescence (ECL) detection kit was from Pierce Biotechnology (Rockford, IL, USA). All the other reagents were from Sigma or as indicated in the specified methods. 

### Animals and treatments

The ICR mice (8-10 week-old; male mice: 28-30 g; female mice: 24-26 g) were purchased from Beijing Vital River whose foundation colonies were all introduced from Charles River Laboratories, Inc (Wilmington, MA, USA). The animals were allowed free access to food and water at all times and were maintained on a 12 h light/dark cycle in a controlled temperature (20-25 °C) and humidity (50 ± 5%) environment for a period of 1 week before use. For mating purposes, four females were housed overnight with two males starting at 9:00 p.m.. Females were checked by 7:00 a.m. the next morning, and the presence of a vaginal plug was designated as gestational day 0 (GD0). 

#### Experiment 1

To investigate the effects of FA on LPS-induced preterm delivery, the pregnant mice were randomly divided into six groups. All pregnant mice except controls (either saline or FA) received an intraperitoneal (i.p.) injection of LPS (300 μg/kg) at 8:00 a.m. on GD15. In LPS+FA groups, the pregnant mice were orally administered with different doses of FA (0.6, 3 or 15 mg/kg, dissolved in 0.3 mL of PBS) 1 h before LPS injection. The 300 μg/kg dose of LPS on GD15 was chosen since preliminary experiment showed that the LPS dosing regimen resulted in 100% dams delivering before GD18. The FA dosing regimen was based on several studies exploring the effects of maternal FA supplementation on pregnancy outcomes in rodents [31,32]. Pregnant mice were observed closely for any signs of preterm delivery (vaginal bleeding, posture). The latency period was defined as the time between LPS or saline solution injection to the delivery of the first pup [7]. 

#### Experiment 2

To investigate the effects of FA on high-dose LPS-induced fetal death, the pregnant mice were treated as experiment 1. All dams were sacrificed 14 h after LPS injection. The dams that exhibited signs of preterm delivery before that time were sacrificed ahead of time. For each litter, the number of live fetuses and dead fetuses was counted. 

#### Experiment 3

To investigate the effects of FA on LPS-induced IUGR, the pregnant mice were randomly divided into six groups. All pregnant mice except controls (either saline or FA) received an i.p. injection of LPS (75 μg/kg) daily from GD15 to GD17. In LPS+FA groups, the pregnant mice were orally administered with different doses of FA (0.6, 3 or 15 mg/kg/d) 1 h before LPS injection. The LPS dosing regimen was based on previous study about LPS-induced IUGR in our lab [8]. All dams were sacrificed on GD18 and gravid uterine weights were recorded. For each litter, the number of resorption sites, dead fetuses and live fetuses was counted. Live fetuses in each litter were weighed and crown-rump lengths were measured. Placentas of live fetuses in each litter were weighted. 

#### Experiment 4

To investigate the effects of FA on LPS-induced inflammation, the pregnant mice were randomly divided into four groups. All pregnant mice except controls (either saline or FA) received an i.p. injection of LPS (300 μg/kg) at 8:00 a.m. on GD15. In LPS+FA group, the pregnant mice were orally administered with 3 mg/kg of FA 1 h before LPS injection. Half of the dams were sacrificed 2 h after LPS injection on time. Placentas were collected at once for measurements of nuclear NF-κB p65. The remaining animals were sacrificed 6 h after LPS injection on time. Maternal serum and amniotic fluid was collected for measurement of interleukin-6 (IL-6), keratinocyte-derived cytokine (KC, murine equivalent of human IL-8) and nitric oxide (NO). Placentas were collected at once for measurements of COX-2. 

This study was approved by the Association of Laboratory Animal Sciences and the Center for Laboratory Animal Sciences at Anhui Medical University (Permit Number: 11-0027). All procedures on animals followed the guidelines for humane treatment set by the Association of Laboratory Animal Sciences and the Center for Laboratory Animal Sciences at Anhui Medical University. 

### Cell culture and treatments

Human trophoblast-like cell line JEG-3 was obtained from the cell bank at the Chinese Academy of Sciences (Shanghai, China) with the original source being the American Type Culture Collection (ATCC) (Manassas, VA, USA). JEG-3 cells were grown in Nunc ﬂasks in Dulbecco’s modiﬁed Eagle’s medium (DMEM) supplemented with 100 U/mL of penicillin, 100 μg/mL streptomycin, 10 mM HEPES, 2 mM L-glutamine, 0.2% NaHCO_3_, and 10% [v/v] heat-inactivated FBS in a humidiﬁed chamber with 5% CO_2_/95% air at 37 °C. The cells were seeded into 6-well culture plates at a density of 5 × 10^5^ cells/well and incubated for at least 12 h to let them adhere to the plates. After being washed three times with medium, the cells were pretreated with FA (40 μg/mL) for 1 h and then stimulated by LPS (2 μg/mL) for 2 h. The FA and LPS dosing regimens were based on previous studies [30,33]. The cells were washed with chilled PBS for three times and then harvested for nuclear protein extraction. 

### Nuclear protein extraction

For nuclear protein extraction from placenta, 400 mg placenta was homogenized in 5 mL ice-cold buffer A [10 mM HEPES (pH 7.9), 150 mM NaCl, 0.6% NP-40, 0.1 mM EDTA, 1 mM dithiothreitol (DDT), and 0.5 mM phenylmethylsulfonyl fluoride (PMSF)] on ice. The homogenate was centrifuged at 270 × *g* for 30 s and the precipitate was discarded. The supernatant was kept on ice for 5 min and centrifuged again at 3,000 × *g* for 20 min at 4 °C. The supernatant was then mixed with 1 mL ice-cold buffer A and centrifuged again at 3,000 × *g* for 5 min. The precipitate containing nuclei was reserved and homogenized in 100 μL Buffer B [20 mM HEPES (pH 7.9), 420 mM NaCl, 1.2 mM MgCl_2_, 25% glycerol, 0.2 mM EDTA, 0.5 mM DDT, 0.5 mM PMSF, 1% Protease Inhbitor Cocktail (P8340, Sigma)] for 60 min on ice. Nuclear lysate was centrifuged at 11,000 × g for 10 min at 4 °C. The supernatant was collected and protein concentrations were determined with the bicinchoninic acid (BCA) protein assay reagents (Pierce, Rockford, IL, USA) according to the manufacturer’s instructions. For nuclear protein extraction from cells, the cells were washed with ice-cold PBS/phosphatase inhibitors, collected with a cell scraper, and harvested by centrifugation. The cell pellet was then resuspended in hypotonic buffer and then kept on ice for 15 min. The suspension was then mixed with detergent and centrifuged for 30 s at 14,000 × g. The nuclear pellet obtained was resuspended in complete lysis buffer in the presence of the protease inhibitor cocktail, incubated for 30 min on ice, and centrifuged for 10 min at 14,000 × g. Protein concentrations were determined with the BCA protein assay reagents. 

### Immunoblot

Total lysate from placenta was prepared by homogenizing 50 mg placenta tissue in 300 μL ice-cold lysis buffer (50 mM Tris-HCl, pH 7.4, 150 mM NaCl, 1 mM EDTA, 1% Triton X-100, 1% sodium deoxycholate, 0.1% sodium dodecyl sulphate, 1 mM PMSF) supplemented with 1% cocktail of protease inhibitors (P8340, Sigma) and centrifuged at 14,000 × g for 10 min at 4 °C. The supernatant was collected and protein concentrations were determined with the BCA protein assay reagents. For immunoblot, same amount of protein (40-80 μg per lane) was separated by SDS-polyacrylamide gel electrophoresis (SDS-PAGE) and transferred onto a polyvinylidene fluoride membrane. The membranes were blocked with 5% defatted milk in Dulbecco phosphate-buffered saline (DPBS) for 2 h at room temperature. The membranes were washed and incubated with 1:1,000 dilutions for 3 h with the following antibodies in commercial primary antibody dilution buffer (Beyotime Institute of Biotechnology, Haimen, China): p-IκB, NF-κB p65 and COX-2. For total protein, β-actin was used as a loading control. For nuclear protein, Lamin A/C was used as a loading control. Alpha-tubulin was used as cytosolic marker. The membranes were washed with DPBS containing 0.05% Tween-20 for 10 min each time, totaling four times. The membranes were then incubated with horse radish peroxidase-linked secondary antibody in 5% defatted milk in DPBS, 1:50,000 dilutions of goat anti-rabbit IgG or goat anti-mouse antibody (Millipore, Billerica, MA, USA) for 2 h. The membranes were washed again and treated with chemiluminescence reagent (ECL; Pierce), and exposed to X-ray film. Relative quantification of each protein was calculated after normalization to loading control protein by densitometric analysis with Image-Pro Plus 6 software. The placentas used for the immunoblot were randomly selected. 

### Immunohistochemistry

Placental tissues were fixed in 4% paraformaldehyde and embedded in paraffin according to the standard procedure. Placental slides (5 μm) were stained with H&E for morphological analysis. Mononuclear sinusoidal trophoblast giant cells in the labyrinth zone can be readily identified on the basis of position and morphology [34]. Sinusoidal trophoblast giant cells have large, round nuclei and can be easily distinguished from other types of cells such as the fetal endothelium or the syncytium. For immunohistochemistry, placental slides were deparaffinized and rehydrated in a graded ethanol series. After antigen retrieval and quenching of endogenous peroxidase, slides were incubated with anti-NF-κB p65 mAb (1:100 dilution) at room temperature for 2 h. The color reaction was developed with an HRP-linked polymer detection system. The stained slides were counterstained with hematoxylin, dehydrated and mounted. Nuclear NF-κB p65 positive cells were counted in six randomly selected fields from each slide at a magnification of × 200. Three cross sections were chosen from each placenta. The number of nuclear NF-κB p65 positive cells was analyzed in six placentas from six pregnant mice. 

### Enzyme-linked immunosorbent assay (ELISA)

Commercial ELISA kits (R&D Systems, Minneapolis, MN, USA) were used to determine the levels of IL-6 and KC in maternal serum and amniotic fluid according to manufacturer's protocol. 

### Analysis of nitric oxide concentration

Nitric oxide (NO) level in maternal serum and amniotic fluid was assessed indirectly by the measurement of its breakdown products, nitrite plus nitrate, using a colorimetric method based on the Griess reaction [35]. Briefly, the samples were incubated with nicotinamide adenine dinucleotide phosphate, flavin adenine dinucleotide, and nitrate reductase for 1 h at 37 °C. Next, lactate dehydrogenase and pyruvate were added, and the samples were further incubated for 30 min at 37 °C. Premixed Griess reagent was then added. After incubation for 10 min at room temperature, the absorbance of each sample was determined at 543 nm. 

### Statistical analysis

The litter was considered the unit for statistical comparison among different groups. Fetal mortality was calculated in litter and then averaged in each group. For fetal weight, crown-rump length, and placental weight, the means were calculated in litter and then averaged in each group. The distribution of the quantitative variables was evaluated by normality test. Normally distributed data were presented as means ± S.E.M.. Statistical analysis of the data was performed using ANOVA and Student-Newmann-Keuls test. Non-normally distributed data were presented as the median and interquartile range (IQR; 25th-75th percentile). Statistical analysis of the data was performed using nonparametric techniques (Kruskal-Wallis test and Mann–Whitney U test). Binomial data were analyzed using χ^2^ analysis or Fisher’s exact test where appropriate. Values of *P* < 0.05 were considered statistically significant. 

## Results

### Effects of FA on LPS-induced preterm delivery

**Figure 1:**
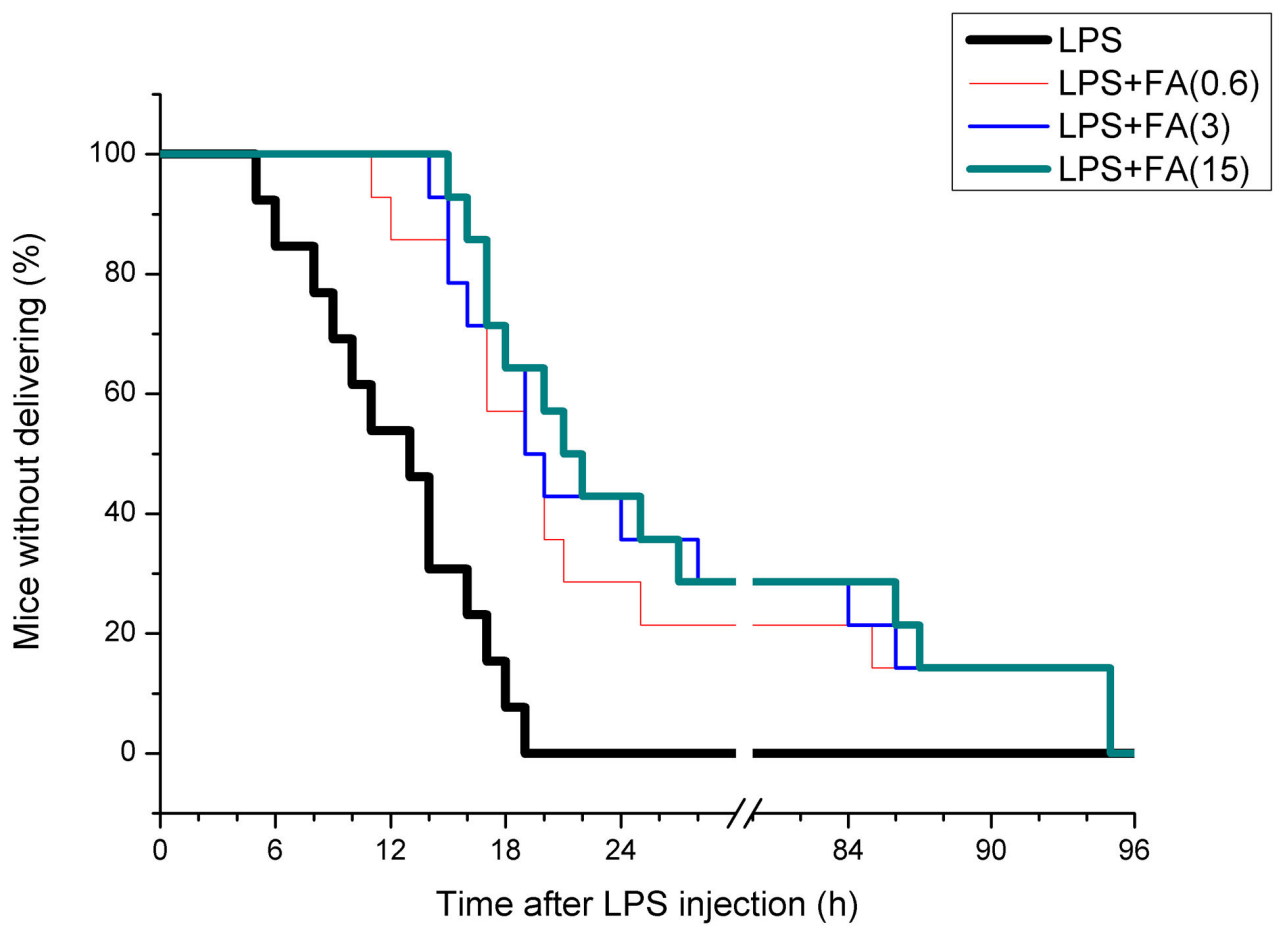
The percentage of mice without delivering after LPS injection. All pregnant mice were i.p. injected with LPS (300 μg/kg) on GD15. In LPS+FA groups, the pregnant mice were orally administered with different doses of FA (0.6, 3 or 15 mg/kg) 1 h before LPS injection. All mice were observed for preterm delivery after LPS injection.

In the saline and FA alone groups, no pregnant mouse delivered before GD18. As shown in Table 1, a high dose of LPS (300 μg/kg) resulted in 100% pregnant mice delivering before GD18. Interestingly, preterm delivery rate dropped to 64.3% when LPS-treated mice were pretreated with high doses of FA (3 or 15 mg/kg), while 71.4% of dams presented preterm delivery when being pretreated with 0.6 mg/kg of FA (Table 1). As shown in Figure 1, the latency interval of preterm delivery was 14 h (IQR: 9.5-17.5 h) in LPS-treated mice. FA pretreatment significantly delayed the latency interval of preterm delivery. The latency interval was 20.5 h (IQR: 16.8-85.8 h) in dams pretreated with 0.6 mg/kg of FA, 22.0 h (IQR: 17.5-86.3 h) in dams pretreated with 3 mg/kg of FA, and 23.5 h (IQR: 18.0-87.3 h) in dams pretreated with 15 mg/kg of FA. 

**Table 1:** Effects of FA on LPS-Induced Preterm Delivery.

	**Control**	**FA**	**LPS**	**LPS+FA (0.6 mg/kg )**	**LPS+FA (3 mg/kg)**	**LPS+FA (15 mg/kg)**
**Number of pregnant mice (n)**	13	13	13	14	14	14
**Litters of preterm delivery (n)**	0	0	13^**^	10	9^‡^	9^‡^
**Incidence of preterm delivery (%)**	0	0	100^**^	71.4	64.3^‡^	64.3^‡^

Note. In the LPS group, the pregnant mice received an i.p. injection of LPS (300 μg/kg) on GD15.

In LPS+FA groups, the pregnant mice were orally administered with different doses of FA (0.6, 3 or 15 mg/kg) 1 h before LPS injection.

^*^ *P* < 0.05, ^**^ *P* < 0.01 VS the control; ‡ *P* < 0.05, ‡‡ *P* < 0.01 VS LPS group.

### Effects of FA on LPS-induced fetal death

**Figure 2:**
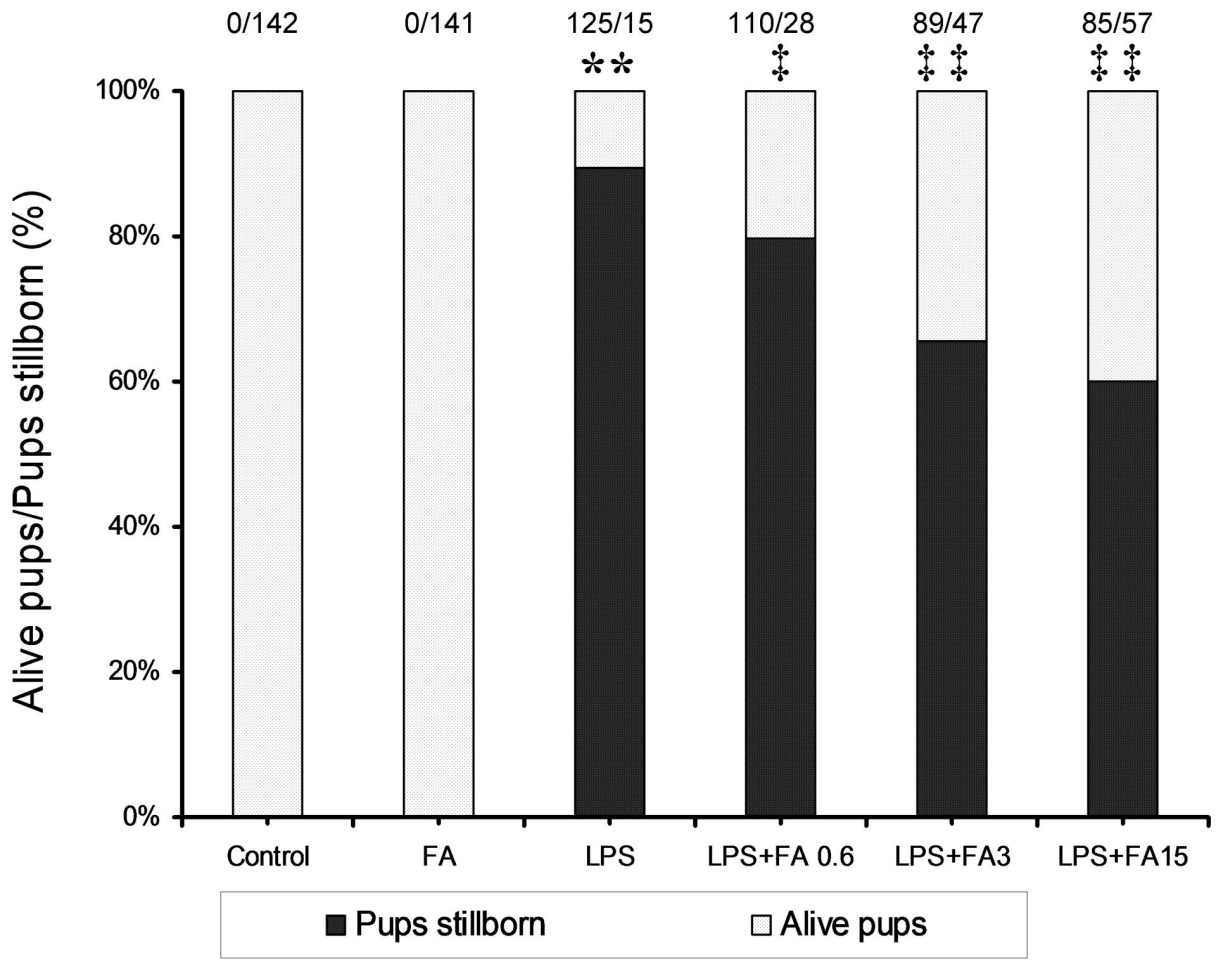
Effects of FA supplementation during pregnancy on high-dose LPS-induced fetal death. All pregnant mice were i.p. injected with LPS (300 μg/kg) on GD15. In LPS+FA groups, the pregnant mice were orally administered with different doses of FA (0.6, 3 or 15 mg/kg) 1 h before LPS injection. Fetal viability was assessed after hysterotomy at 14 h after LPS injection. The number of dams analyzed is 10 in each group. The fraction on the top of the bar charts refers to the total number of dead fetuses/number of live fetuses in each group. ** *P* < 0.01 VS the control. ‡ *P* < 0.05, ‡‡ *P* < 0.01 VS LPS group.

The effects of FA on high-dose LPS-induced fetal death are presented in Figure 2. Fourteen hours after a high dose of LPS (300 μg/kg) injection, 89% of fetuses were dead in LPS-treated mice. As shown in Table 2, injection with a low dose of LPS (75 μg/kg) daily resulted in 9 % (1/11) of dams delivering before GD18. In addition, 29.3% of fetuses were dead in dams that completed the pregnancy. Interestingly, FA supplementation during pregnancy significantly alleviated LPS-induced fetal death in a dose-dependent manner (Table 2 and Figure 2). 

**Table 2:** Effects of FA on LPS-Induced IUGR.

	**Control**	**FA**	**LPS**	**LPS+FA (0.6 mg/kg )**	**LPS+FA (3 mg/kg)**	**LPS+FA (15 mg/kg)**
**Number of pregnant mice (n)**	11	11	11	11	11	11
**Litters of preterm delivery** (**n**)	0	0	1	1	0	0
**Litters of term delivery** (**n**)	11	11	10	10	11	11
**Dead fetuses per litter (n)^*a*^**	0.18±0.12	0.09±0.09	4.10±1.22^**^	3.10±1.18	1.18±0.42^‡^	0.91±0.31^‡^
**Live fetuses per litter (n**) ^a^	13.09±0.71	13.73±0.43	10.56±1.27	11.67±0.58	11.82±0.67	12.09±0.68
**Fetal weight** (**g**)	1.39±0.02	1.39±0.01	1.16±0.02^**^	1.26±0.03^‡‡^	1.24±0.02^‡‡^	1.30±0.02^‡‡^
**Crown-rump length** (**cm**)	2.45±0.01	2.47±0.01	2.34±0.03^**^	2.38±0.02	2.38±0.01	2.41±0.02^‡^
**Average placental weight (g)**	1.101±0.002	1.102±0.002	0.088±0.003^**^	0.091±0.003	0.090±0.003	0.094±0.004

Note. In the LPS group, the pregnant mice received an i.p. injection of LPS (75 μg/kg/d) on GD15-GD17.

In LPS+FA groups, the pregnant mice were orally administered with different doses of FA (0.6, 3 or 15 mg/kg/d) 1 h before LPS injection.

All quantitative data were presented as means ± SEM.

^a^ The number of dead or live fetuses per litter in dams that completed the pregnancy.

* P < 0.05, ** P < 0.01 VS the control; ‡ P < 0.05, ‡‡ P < 0.01 VS LPS group.

### Effects of FA on LPS-induced IUGR

As shown in Table 2, LPS injection markedly reduced the weight and crown-rump length of live fetuses. FA supplementation during pregnancy had no effect on fodder consumption and weight gain of the pregnant mice (data not shown). Interestingly, maternal FA supplementation significantly alleviated LPS-induced reduction of fetal weight and crown-rump length in a dose-dependent manner (Table 2). 

### Effects of FA on LPS-induced NF-κB activation in mouse placentas

**Figure 3:**
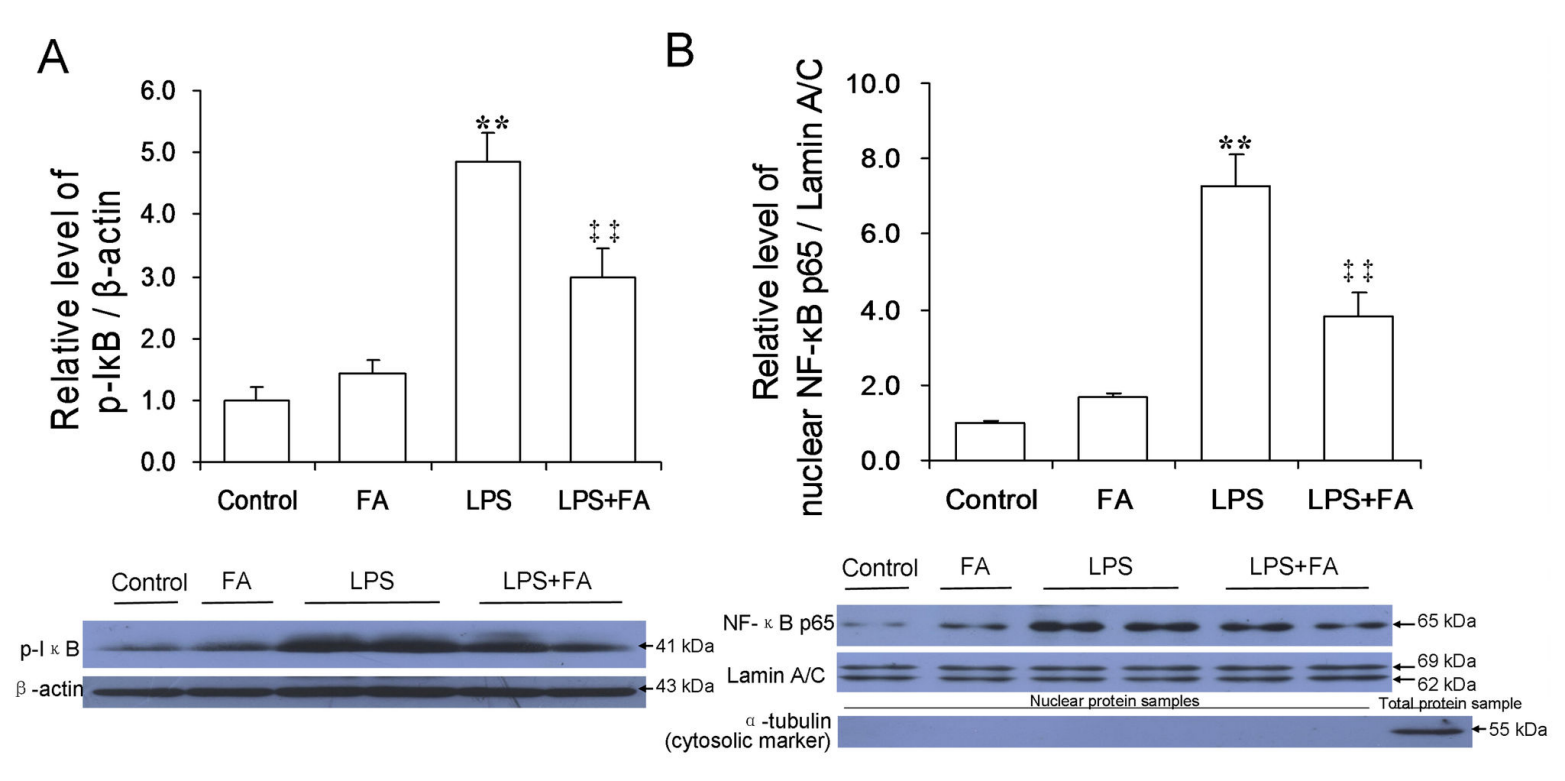
FA inhibits LPS-induced NF-κB activation in mouse placenta. All pregnant mice were i.p. injected with LPS (300 μg/kg) on GD15. In LPS+FA groups, the pregnant mice were orally administered with 3 mg/kg of FA 1 h before LPS injection. Placentas were collected 2 h after LPS injection. The phosphorylation of IκB and nuclear NF-κB p65 was determined by immunoblot. (A) Placental p-IκB. (B) Nuclear NF-κB p65 in placentas. All experiments were repeated for three times. All data were presented as means ± S.E.M. (control n=3, FA n=3, LPS n=6, LPS+FA n=6) ** *P* < 0.01 VS the control. ‡‡ *P* < 0.01 VS LPS group.

**Figure 4:**
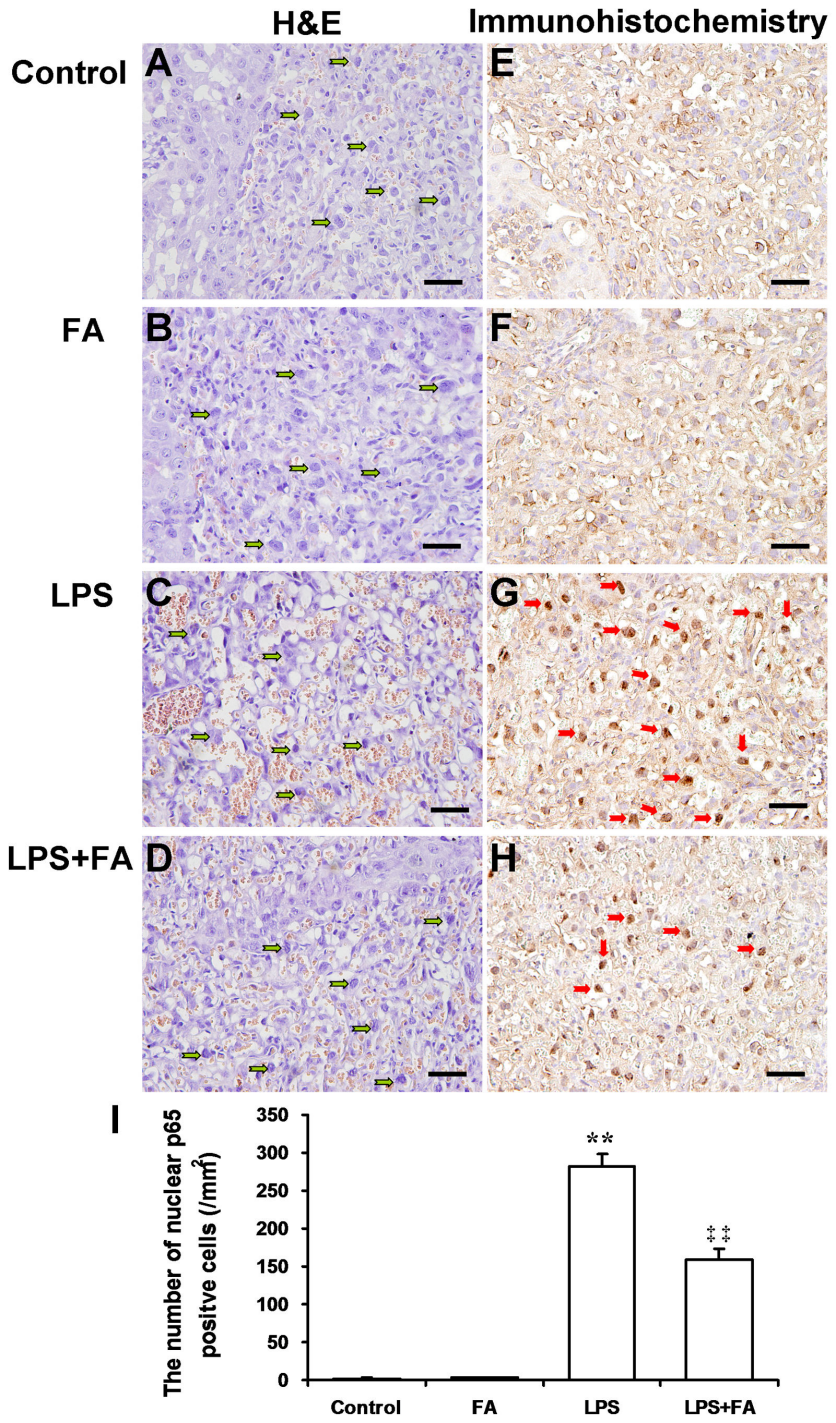
Effects of FA on LPS-induced nuclear translocation of NF-κB p65 in mouse placenta. All pregnant mice were i.p. injected with LPS (300 μg/kg) on GD15. In LPS+FA groups, the pregnant mice were orally administered with 3 mg/kg of FA 1 h before LPS injection. Placentas were collected 2 h after LPS injection. (A–D) Representative photomicrographs of placental histological specimens from mice treated with saline (A as control), FA alone (B), LPS alone (C), and LPS+FA (D) are shown (H&E). Mononuclear sinusoidal trophoblast giant cells were distributed in the labyrinth zone (green arrowheads). Original magnification, × 200. Scale bars 50 µm. (E–H) Nuclear translocation of NF-κB p65 was analyzed using immunohistochemistry. Representative photomicrographs of placental histological specimens from mice treated with saline (E as control), FA alone (F), LPS alone (G), and LPS+FA (H) are shown. Nuclear translocation of NF-κB p65 was observed in mononuclear sinusoidal trophoblast giant cells of the labyrinth zone (red arrowheads). Original magnification, × 200. Scale bars 50 µm. (I) The number of nuclear NF-κB p65 positive cells per square millimeter (mm^2^). All data were presented as means ± S.E.M. (n=6 mice per group) ** *P* < 0.01 VS the control. ‡‡ *P* < 0.01 VS LPS group.

As shown in Figure 3, the level of phosphorylated IκB was significantly increased in placentas of mice injected with LPS. Correspondingly, the level of nuclear NF-κB p65 was significantly increased in placentas of LPS-treated mice. Remarkably, FA pretreatment significantly attenuated LPS-induced translocation of NF-κB p65 to the nuclei (Figure 3A and 3B). Immunohistochemistry showed that LPS-induced nuclear translocation of NF-κB p65 was mainly observed in mononuclear sinusoidal trophoblast giant cells of the labyrinth zone (Figure 4G and 4I). FA pretreatment inhibited LPS-induced nuclear translocation of NF-κB p65 in mononuclear sinusoidal trophoblast giant cells of the labyrinth zone (Figure 4G, 4H and 4I). In addition, H&E stain showed that LPS injection caused obvious hyperemia in placental labyrinth zone. Interestingly, pretreatment with FA attenuated LPS-induced hyperemia in mouse placentas (Figure 4A-4D). 

### Effects of FA on LPS-induced NF-κB activation in human placental trophoblastic-derived cells

**Figure 5:**
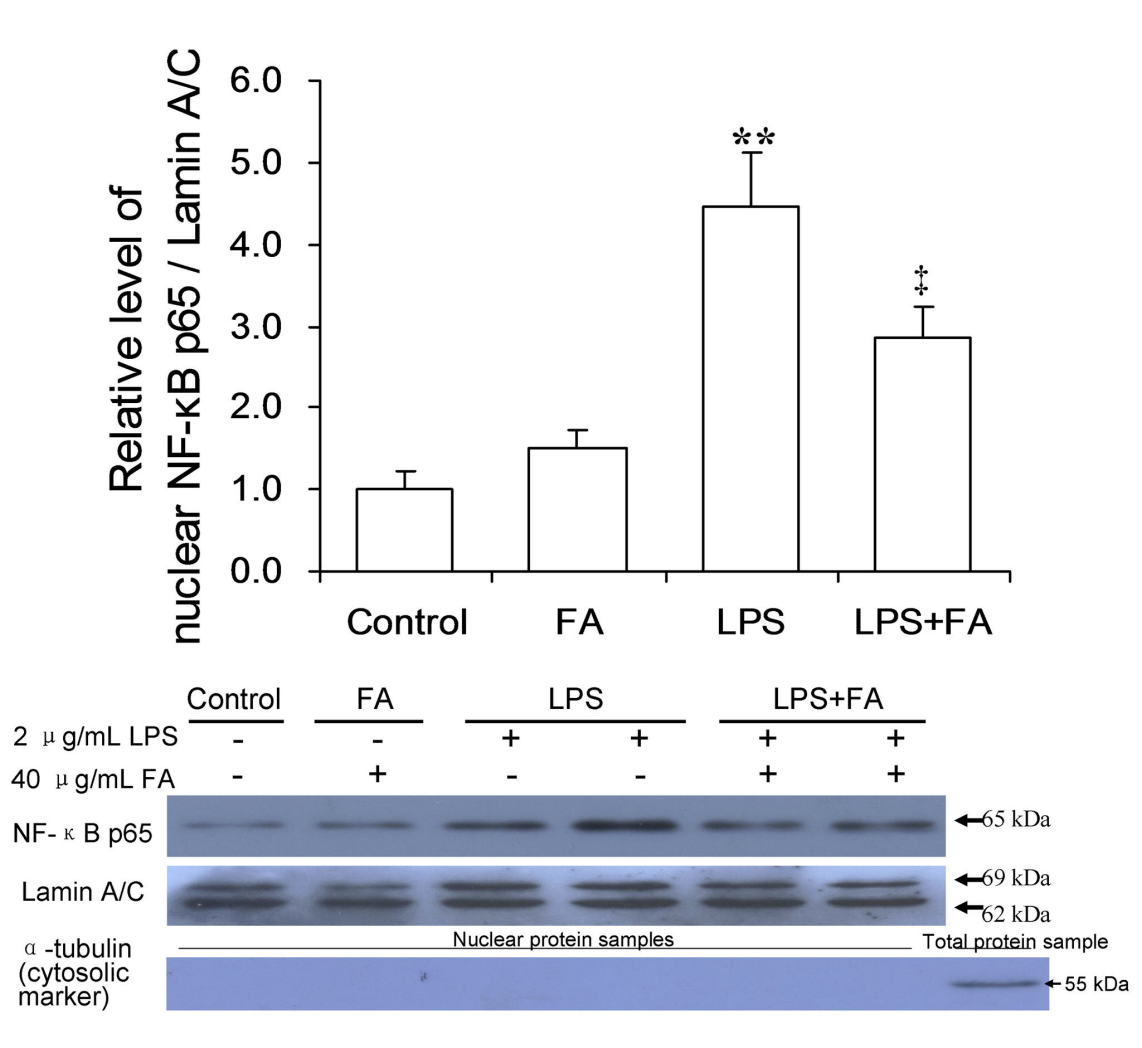
Effects of FA on LPS-induced nuclear translocation of NF-κB p65 in human JEG-3 cells. Human trophoblast-derived cells (JEG-3 cells) were pretreated with FA (40 μg/mL) for 1 h and then stimulated by LPS (2 μg/mL) for 2 h. Nuclear NF-κB p65 was determined by immunoblot. The results are representative of three independent experiments and presented as means ± S.E.M.. ** *P* < 0.01 VS the control. ‡ *P* < 0.05 VS LPS group.

Next, we tested whether FA inhibits LPS-induced NF-κB activation in human placental trophoblast cell line JEG-3. As shown in Figure 5, LPS significantly increased the level of nuclear NF-κB p65 in human JEG-3 cells. Interestingly, pretreatment with FA significantly alleviated LPS-stimulated NF-κB activation in human JEG-3 cells (Figure 5). 

### Effects of FA on LPS-induced upregulation of COX-2

**Figure 6:**
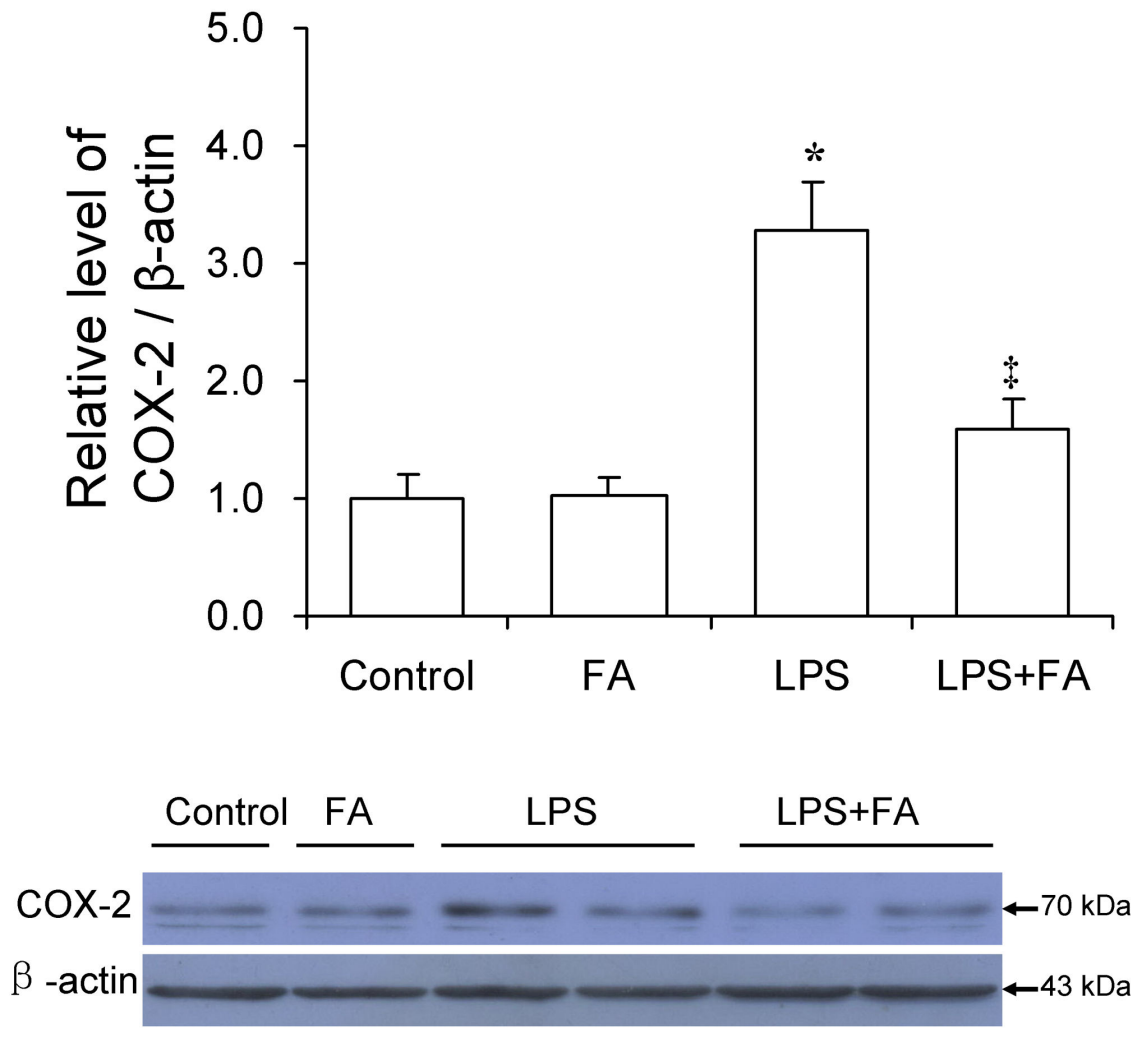
FA inhibits LPS-induced placental COX-2 expression. All pregnant mice were i.p. injected with LPS (300 μg/kg) on GD15. In LPS+FA groups, the pregnant mice were orally administered with 3 mg/kg of FA 1 h before LPS injection. Placentas were collected 6 h after LPS injection. COX-2 was determined by immunoblot. All experiments were repeated for three times. All data were presented as means ± S.E.M. (control n=3, FA n-3, LPS n=6, LPS+FA n=6) * *P* < 0.05 VS the control. ‡ *P* < 0.05 VS LPS group.

As shown in Figure 6, LPS significantly increased the level of placental COX-2. Interestingly, FA pretreatment significantly attenuated LPS-induced upregulation of COX-2 in placentas. 

### Effects of FA on LPS-induced release of inflammatory cytokines

**Figure 7:**
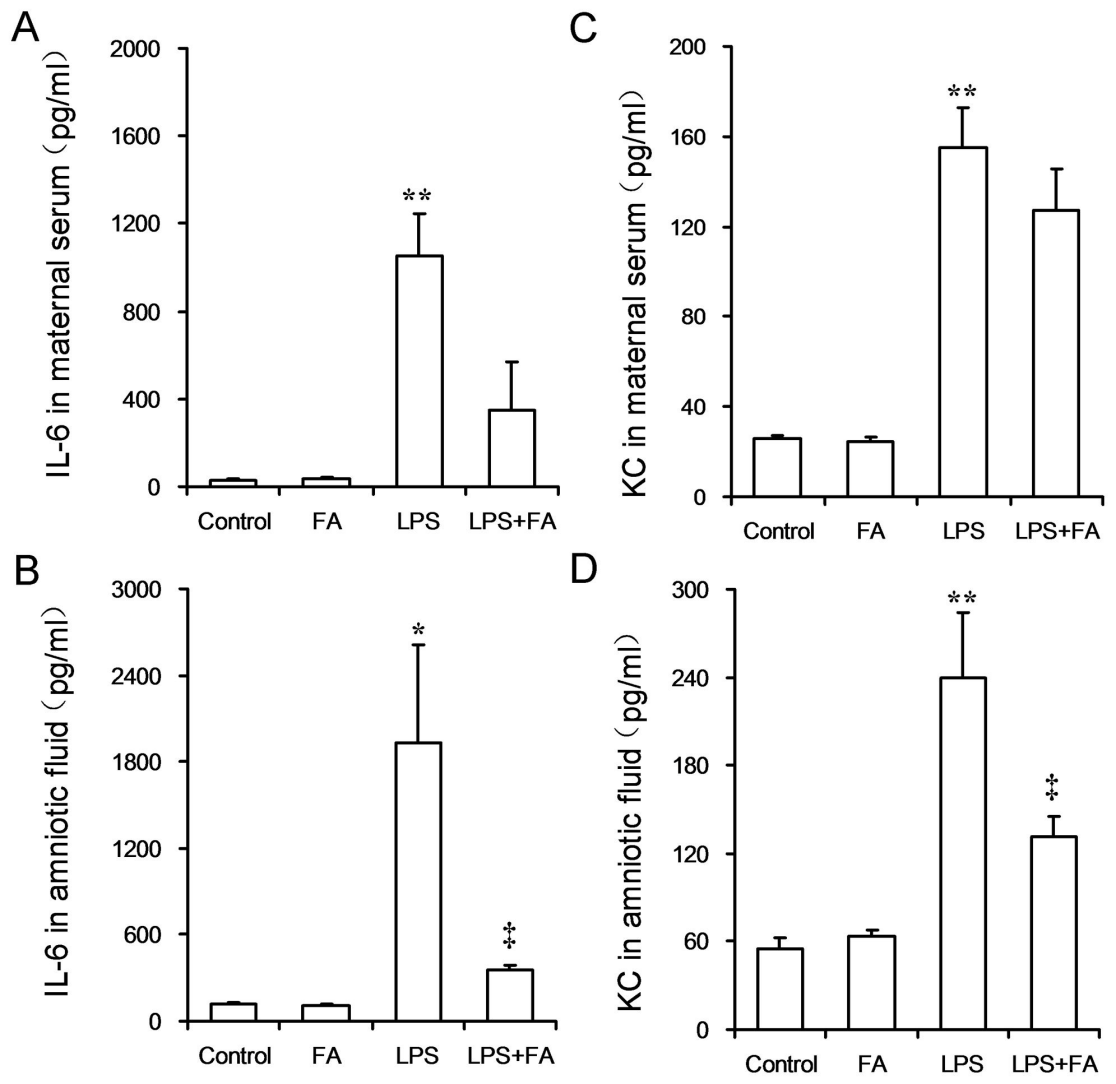
FA inhibits LPS-evoked inflammatory cytokines in pregnant mice. All pregnant mice were i.p. injected with LPS (300 μg/kg) on GD15. In LPS+FA groups, the pregnant mice were orally administered with 3 mg/kg of FA 1 h before LPS injection. (A and B) Maternal serum was collected 6 h after LPS injection. The levels of IL-6 and KC in maternal serum were measured using ELISA. (A) IL-6. (B) KC. (C and D) Amniotic fluid was collected 6 h after LPS injection. The levels of IL-6 and KC in amniotic fluid were measured using ELISA. (C) IL-6. (D) KC. All data were presented as means ± S.E.M (n=6 mice per group). * *P* < 0.05, ** *P* < 0.01 VS the control. ‡ *P* < 0.05 VS LPS group.

As shown in Figures 7A and 7B, the level of IL-6 was significantly increased in maternal serum and amniotic fluid of LPS-treated mice. Interestingly, the level of KC, a murine equivalent of human IL-8, was significantly increased in maternal serum and amniotic fluid of LPS-treated mice (Figures 7C and 7D). FA pretreatment significantly alleviated LPS-induced release of IL-6 in amniotic fluid (Figure 7B). In addition, FA pretreatment significantly alleviated LPS-induced release of KC in amniotic fluid (Figure 7D). 

### Effects of FA on LPS-induced NO production

**Figure 8:**
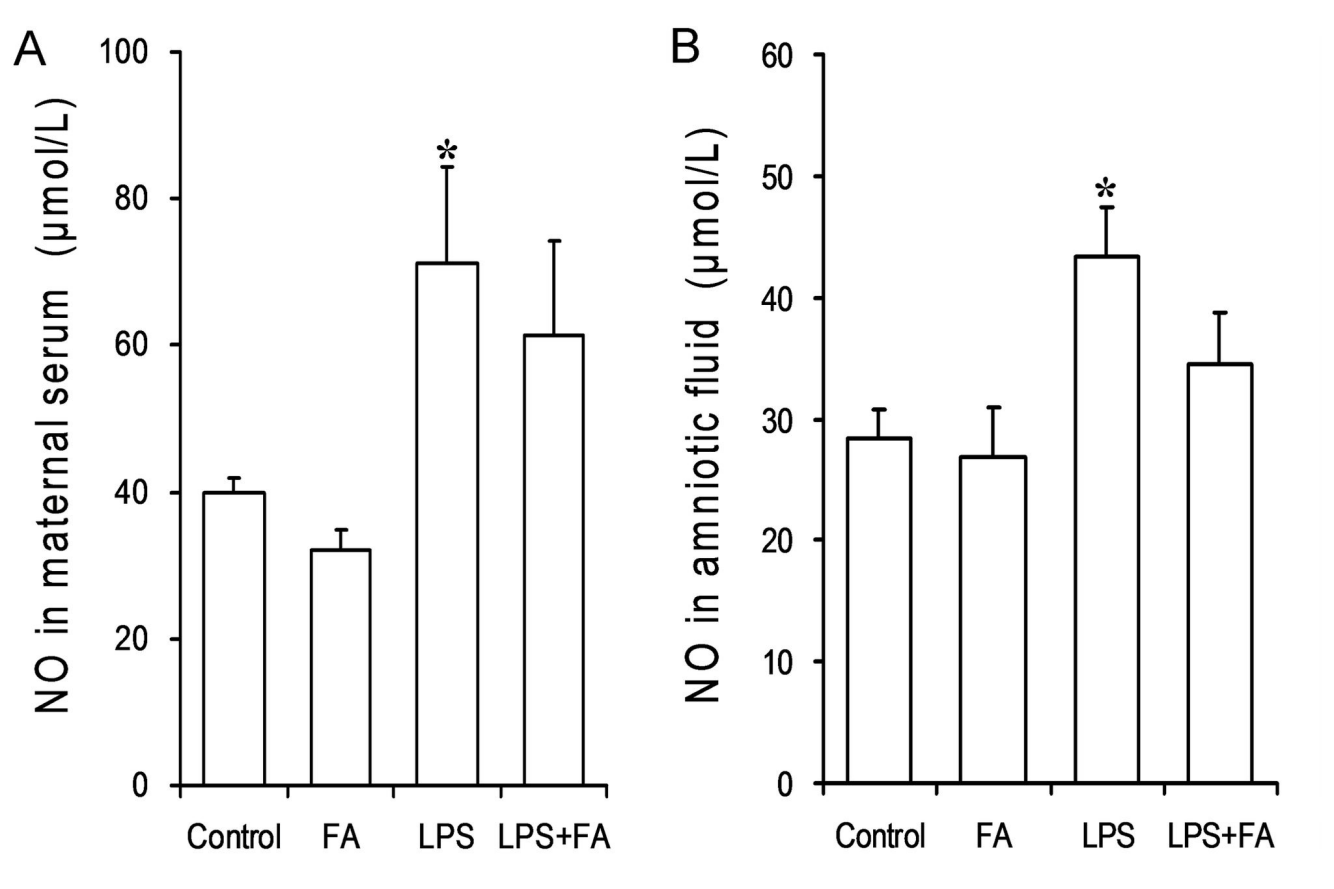
Effects of FA on LPS-induced NO production in pregnant mice. All pregnant mice were i.p. injected with LPS (300 μg/kg) on GD15. In LPS+FA groups, the pregnant mice were orally administered with 3 mg/kg of FA 1 h before LPS injection. Maternal serum and amniotic fluid were collected 6 h after LPS injection. Nitrite plus nitrate concentrations were measured in maternal serum and amniotic fluid. Data were presented as means ± S.E.M (n=6 mice per group). *P < 0.05 VS the control.

To verify the effects of FA on LPS-evoked NO production, nitrite plus nitrate concentration in maternal serum and amniotic fluid was measured. As expected, nitrite plus nitrate concentration was significantly increased in maternal serum and amniotic fluid of LPS-treated mice. However, FA pretreatment had little effect on LPS-induced release of NO in maternal serum and amniotic fluid (Figure 8). 

## Discussion

Much evidence demonstrates that physiological supply of FA (0.4-0.8 mg/d) during pregnancy can reduce the incidence of FA deficiency-related NTDs [36]. Recently, studies show that the beneficial effects of maternal FA supplementation may extend beyond NTDs [37]. Two cohort studies showed that supplementation of high-dose FA (3.7-5 mg/d) reduced the risk of preterm delivery and low birth weight [27,28]. However, whether maternal FA supplementation prevents preterm delivery and IUGR remains controversial [38]. In the present study, we investigated the effects of supplementation with a high dose of FA on LPS-induced preterm delivery, fetal death and IUGR in mice. The results showed that FA delayed the latency interval of preterm delivery and reduced the incidence of preterm delivery. Moreover, FA supplementation during pregnancy significantly reduced the number of dead fetuses per litter in LPS-treated mice. In addition, FA significantly attenuated LPS-induced IUGR. These results suggest that FA might play a part in preventing bacterial infect-related preterm delivery and IUGR. 

Indeed, inflammatory cytokines are involved in LPS-induced preterm delivery and IUGR [8,21,39-41]. Increasing evidence indicates that FA has an anti-inflammatory effect. Two intervention trials showed that FA supplementation decreased the circulating level of several inflammatory mediators in healthy overweight subjects and in patients with inflammatory bowel diseases [42,43]. A recent study found that FA inhibited LPS-stimulated inflammatory cytokines in macrophages [30]. Furthermore, a recent study suggested that FA status during pregnancy might act by rectifying genital tract inflammatory milieu to reduce the risk of preterm delivery [29]. The present study further investigated the effects of FA pretreatment on LPS-induced release of inflammatory cytokines in maternal serum and amniotic fluid. The results showed that FA pretreatment significantly alleviated LPS-induced elevation of IL-6 in amniotic fluid. Moreover, FA pretreatment significantly attenuated LPS-induced release of KC, the murine equivalent of human IL-8, in amniotic fluid. These results suggest that the protection of FA against LPS-induced preterm delivery and IUGR might, at least partially, be attributed to its anti-inflammatory effects. 

According to an earlier study, cyclooxygenase (COX)-2-mediated PGs production is a key pathophysiologic event in LPS-induced fetal death [17]. Further studies showed that COX-2 suppressors prevented LPS-induced fetal death and preterm delivery [18,19]. Indeed, the present study showed that placental COX-2 expression was significantly upregulated in LPS-treated mice. Interestingly, we found for the first time, to our knowledge, that maternal FA pretreatment significantly attenuated LPS-induced upregulation of COX-2 in placenta. The finding suggests that FA-mediated protection against LPS-induced preterm delivery, fetal death and IUGR might be due to its repression of COX-2 expression in placenta. 

NF-κB activation plays a central role in LPS-evoked expression of inflammatory cytokines and COX-2 [44]. Under unstimulated conditions, NF-κB is usually retained in the cytoplasm by binding to I-κB. I-κB phosphorylation causes translocation of NF-κB to the nucleus [45]. The present study showed that the level of placental phosphorylated I-κB was significantly increased in LPS-treated pregnant mice. Correspondingly, the level of nuclear NF-κB p65 was significantly increased in the placentas of mice injected with LPS. Immunohistochemistry showed that nuclear translocation of NF-κB p65 was mainly observed in mononuclear sinusoidal trophoblast gaint cells of the labyrinth zone. Correspondingly, incubation of human JEG-3 cells with LPS significantly increased the level of nuclear NF-κB p65. A recent *in vitro* study showed that FA abrogated LPS-induced NF-κB activation in macrophages [30]. In the current study, we found that FA significantly attenuated LPS-evoked placental I-κB phosphorylation and LPS-induced elevation of nuclear NF-κB p65 in the murine placenta. The present study showed that folic acid significantly attenuated LPS-evoked placental I-κB phosphorylation. Moreover, folic acid pretreatment significantly inhibited LPS-induced translocation of NF-κB p65 to nucleus in the murine placenta. Importantly, FA inhibited LPS-induced nuclear translocation of NF-κB p65 in mononuclear sinusoidal trophoblast giant cells of the labyrinth zone. Furthermore, FA suppressed LPS-induced translocation of NF-κB p65 to the nucleus in human trophoblast cell line JEG-3. According to an *in vitro* study, LPS stimulated NF-κB activation that corresponded with upregulation of COX-2 protein expression and IL-6 release in human JEG-3 cells [46]. Collectively, these results suggest that maternal FA supplementation during pregnancy suppresses LPS-induced release of inflammatory cytokines and COX-2 expression through its inhibition of placental NF-κB activation. 

Although NO fulfills important functions during pregnancy, it has toxic effects at high concentrations such as those produced in sepsis [16,47]. According to a recent study, aminoguanidine, a selective inhibitor of iNOS, prevented LPS-induced preterm labor [48]. Indeed, an *in vitro* study showed that FA significantly inhibited NO production in LPS-stimulated RAW264.7 cells [30]. In the present study, we investigated whether maternal FA supplementation during pregnancy could block LPS-evoked NO production in maternal serum and amniotic fluid. As expected, the level of nitrite plus nitrate, the stable end metabolites of NO, was significantly increased in maternal serum and amniotic fluid of mice treated with LPS. However, FA had little effect on LPS-induced release of NO in maternal serum and amniotic fluid. These results suggest that protective effect of FA on LPS-induced preterm delivery and IUGR may be independent of its inhibition of NO production. 

In summary, the present study indicates that maternal FA supplementation during pregnancy protects against LPS-induced preterm delivery, fetal death and IUGR in mice. We demonstrate for the first time, to our knowledge, that FA-mediated protection against LPS-induced preterm delivery, fetal death and IUGR might, at least partially, be attributed to its anti-inflammatory effects. Thus, FA may be used in the future as anti-inflammatory agent in combination with antibiotics to protect against inflammation-related preterm delivery and IUGR. 
